# Vascular amyloid accumulation alters the gabaergic synapse and induces hyperactivity in a model of cerebral amyloid angiopathy

**DOI:** 10.1111/acel.13233

**Published:** 2020-09-10

**Authors:** Pablo Cisternas, Xavier Taylor, Abigail Perkins, Orlando Maldonado, Elysabeth Allman, Ricardo Cordova, Yamil Marambio, Braulio Munoz, Taylor Pennington, Shunian Xiang, Jie Zhang, Ruben Vidal, Brady Atwood, Cristian A. Lasagna‐Reeves

**Affiliations:** ^1^ Stark Neurosciences Research Institute Indiana University School of Medicine Indianapolis IN USA; ^2^ Department of Anatomy, Cell Biology & Physiology Indiana University School of Medicine Indianapolis IN USA; ^3^ Department of Pharmacology & Toxicology Indiana University School of Medicine Indianapolis IN USA; ^4^ Department of Medical and Molecular Genetics Indiana University School of Medicine Indianapolis IN USA; ^5^ Department of Pathology and Laboratory Medicine Indiana University School of Medicine Indianapolis IN USA

**Keywords:** cerebral amyloid angiopathy, GABAergic synapses, synaptotoxicity, vascular amyloid

## Abstract

Cerebral amyloid angiopathy (CAA) is typified by the cerebrovascular deposition of amyloid. The mechanisms underlying the contribution of CAA to neurodegeneration are not currently understood. Although CAA is highly associated with the accumulation of β‐amyloid (Aβ), other amyloids are known to associate with the vasculature. Alzheimer's disease (AD) is characterized by parenchymal Aβ deposition and intracellular accumulation of tau as neurofibrillary tangles (NFTs), affecting synapses directly, leading to behavioral and physical impairment. CAA increases with age and is present in 70%–97% of individuals with AD. Studies have overwhelmingly focused on the connection between parenchymal amyloid accumulation and synaptotoxicity; thus, the contribution of vascular amyloid is mostly understudied. Here, synaptic alterations induced by vascular amyloid accumulation and their behavioral consequences were characterized using a mouse model of Familial Danish dementia (FDD), a neurodegenerative disease characterized by the accumulation of Danish amyloid (ADan) in the vasculature. The mouse model (Tg‐FDD) displays a hyperactive phenotype that potentially arises from impairment in the GABAergic synapses, as determined by electrophysiological analysis. We demonstrated that the disruption of GABAergic synapse organization causes this impairment and provided evidence that GABAergic synapses are impaired in patients with CAA pathology. Understanding the mechanism that CAA contributes to synaptic dysfunction in AD‐related dementias is of critical importance for developing future therapeutic interventions.

## Graphical abstract

In healthy individuals, normal synaptic organization and function is preserved by the gliovascular unit (GVU). Under cerebral amyloid angiopathy (CAA), vascular amyloid accumulation (1) causes an impairment in the GVU. This in turn causes inhibitory synapse (IS) damage (2), causing a disorganization of its structure (3). As a result, components of the IS are co‐deposited with amyloid in the vasculature (4). Therefore, vascular amyloid pathology likely plays a significant role in synaptic impairment.

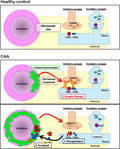

## 1 INTRODUCTION

Alzheimer's disease (AD) is responsible for the majority of age‐related progressive memory loss and dementia. AD is a multifactorial neurodegenerative disease for which no effective therapies currently exist (Long & Holtzman, 2019). It is characterized by extensive parenchymal β‐amyloid (Aβ) accumulation and intracellular accumulation of tau as neurofibrillary tangles (NFTs) that have been directly linked to neuropathological events such as loss of synapses and neuronal cells (Long & Holtzman, 2019). Several studies have associated cerebrovascular pathologies with an increased risk for the development of AD and other dementias, indicating that the pathological mechanisms present during vascular disturbance contribute to the progression of the disease (Snyder et al., 2015). Cerebral amyloid angiopathy (CAA), typified by the cerebrovascular deposition of amyloid, is one of the most prevalent pathologies associated with AD (Cisternas, Taylor, & Lasagna‐Reeves, 2019). CAA is present in an estimated 70%–97% of individuals with AD (Attems, 2005) and directly increases with age. Additionally, CAA is present in 36%, 46%, and 58%–99% of individuals over 60, 70, and 90, respectively (Attems, 2005; Holton et al., 2002; Vinters & Gilbert, 1983), positioning CAA as a strong vascular contributor to AD and age‐related cognitive decline. In CAA, amyloid is thought to originate in the neurons and is drained along the perivascular interstitial fluid pathway of the brain parenchyma and leptomeninges, depositing in blood vessels (Weller, Subash, Preston, Mazanti, & Carare, 2008). This amyloid accumulation progresses until the blood vessel is severely damaged, altering the blood–brain barrier and the gliovascular unit, leading to detrimental glial activation, neuroinflammation, and neurodegeneration (Cisternas et al., 2019).

Synapses connect neurons to form a vast network to facilitate behavioral and cognitive processes, such as thinking and memory. Excitatory and inhibitory synapses play a specific coordinated and tightly regulated role in these processes (Tao et al., 2018). Consistent synaptic failure and loss is a major characteristic of AD and the primary contributor to cognitive impairment in affected individuals (Arendt, 2009). Synapses are regarded as the earliest site of pathology, and synaptic dysfunction is observed before neuronal loss and coincides with the onset of memory and cognitive deficits (Rudy, Hunsberger, Weitzner, & Reed, 2015). Quantitative ultrastructural studies on brains from individuals with AD revealed a 25%–35% decrease in the overall density of synapses and a 15%–35% synaptic loss per neuron (Davies, Mann, Sumpter, & Yates, 1987). Synaptic changes localize with amyloid accumulation (Koffie, Hyman, & Spires‐Jones, 2011). The majority of studies have focused on the connection between parenchymal amyloid accumulation and synaptotoxicity (Jackson et al., 2019); thus, little is known about the contribution of vascular amyloid to AD progression.

Although CAA is highly associated with the accumulation of Aβ (Attems, 2005), other types of amyloids associate with the vasculature. Therefore, CAA could be considered as a group of biochemically and genetically diverse disorders unified by amyloid accumulation in the arterial blood vessel walls (Cisternas et al., 2019). Two examples of non‐Aβ CAA are Familial British dementia (FBD) and Familial Danish dementia (FDD), where a mutated form of integral membrane protein 2B (BRI2) causes vascular and leptomeningeal accumulation of the ABri or ADan amyloid, respectively (Garringer, Murrell, D'Adamio, Ghetti, & Vidal, 2010). FDD is characterized by the presence of CAA due to the accumulation of ADan in the leptomeninges and the vessels of the gray and white matter. Genetic analysis of FDD patients identified a 10‐nucleotide insertion at the 3′‐end of the BRI2 coding region. This frameshift mutation produces the 277‐amino‐acid ADan precursor protein, of which the ~4 kDa Danish amyloid subunit comprises the last 34 amino acids (Garringer et al., 2010). Cotton wool‐like plaques in the vicinity of blood vessels with amyloid and tau NFTs are also observed in FDD patients (Holton et al., 2002). A mouse model for FDD (Tg‐FDD) (Vidal, Barbeito, Miravalle, & Ghetti, 2009) consistently exhibits CAA, primarily in leptomeningeal cerebellar vessels (Vidal et al., 2009) and in large‐ and medium‐sized parenchymal and penetrating vessels of the brain. Perivascular tau immunoreactive deposits have also been observed in Tg‐FDD mice (You et al., 2019), yet the relationship between vascular amyloid deposits and synaptotoxicity has not been established. Thus, Tg‐FDD mice are a valuable model for characterizing CAA‐associated synaptic dysfunction.

In the present study, we analyzed the contributions of CAA to synaptic dysfunction in the Tg‐FDD model. As CAA is prevalent in the elderly population, we analyzed 18‐month‐old mice. This age meets the definition of “elderly” in which senescent changes are observed in almost all biomarkers (Dutta & Sengupta, 2016). We found that Tg‐FDD mice exhibit a hyperactive phenotype coinciding with significant alterations in inhibitory synaptic markers. Interestingly, vascular amyloid deposits were accompanied by some of these inhibitory synaptic markers. The excitatory synapse components remained unaltered in these mice. We complemented our investigation by analyzing publicly available RNA‐Seq data from human brain samples with CAA pathology, revealing significant alterations in inhibitory synaptic pathways, supporting the observations from the Tg‐FDD model. Our study indicates that vascular amyloid accumulation contributes to synaptic damage, expanding the understanding of the processes that lead to cognitive decline in neurodegenerative dementias with cerebrovascular pathologies.

## 2 RESULTS

### 2.1 Tg‐FDD mice exhibit hyperactive behavior.

**FIGURE 1:**
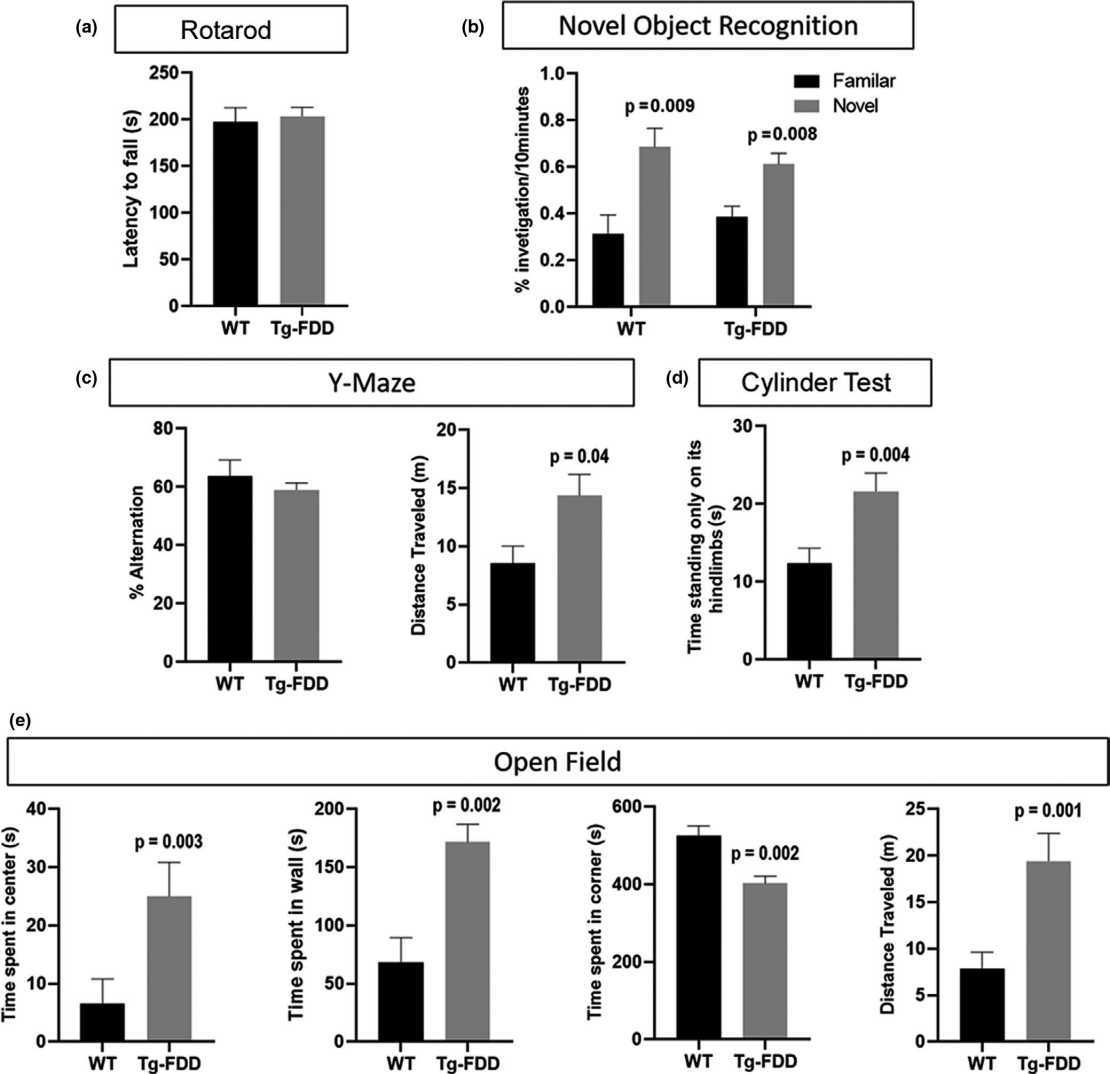
Hyperactive phenotype in Tg‐FDD mice. Mice were subjected to different behavioral tests to assess motor and cognitive functions and overall activity. (A) No motor deficits in Tg‐FDD were observed in the rotarod assay. (B, C) Tg‐FDD did not show signs of cognitive impairment in the NOR and Y‐Maze assay. An increase in distance traveled by Tg‐FDD was observed in the Y‐Maze. (D) Tg‐FDD showed an increase in spontaneous activity measured in the Cylinder test assay. (E) In the open‐field assay, Tg‐FDD showed an increase in time spent in the center and walls of the arena but a decrease in time in the corners in relation to WT. An increase in distance traveled by Tg‐FDD was also observed. *p* < 0.05 is indicated on each graph. Mann–Whitney test, *n* = 12–16 animals per genotype. Data represented as mean + SEM.

As synaptic damage is associated with cognitive deficits and altered behavior (Lepeta et al., 2016), we subjected 18‐month‐old Tg‐FDD and wild‐type (WT) mice to a battery of behavioral tests (Figure 1). When subjected to rotarod assay, no difference was observed between Tg‐FDD and WT mice, suggesting no motor impairment in this model (Figure 1A). Tg‐FDD mice did not differ from WT mice in the novel object recognition tests (Figure 1B), indicating no impairment in cognitive functioning or memory. No differences were observed between Tg‐FDD and WT on the percent of alternation in the Y‐Maze, confirming normal cognitive function in Tg‐FDD mice (Figure 1C). Interestingly, Tg‐FDD mice traveled a longer distance in the Y‐Maze in total (Figure 1C), suggesting a hyperactive phenotype. Therefore, we compared the spontaneous activity of Tg‐FDD and WT mice using the Cylinder test (Fleming, Ekhator, & Ghisays, 2013). Remarkably, Tg‐FDD mice spent more time in vertical movement, standing on hindlimbs with both forelimbs off the floor, than WT mice (Figure 1D), further indicating hyperactivity or an enhancement of sensorimotor function (Fleming et al., 2013). Finally, we measured general locomotor activity with the open‐field test. Tg‐FDD mice statistically spent more time in the center and in the walls of the open field compared with WT mice and less time in the corners (Figure 1E). The total distance traveled by Tg‐FDD mice was again significantly higher than WT mice (Figure 1E). Overall, these behavioral analyses indicate that the Tg‐FDD mice have a hyperactive phenotype.

### 2.2 Alteration of inhibitory synapse in Tg‐FDD

**FIGURE 2:**
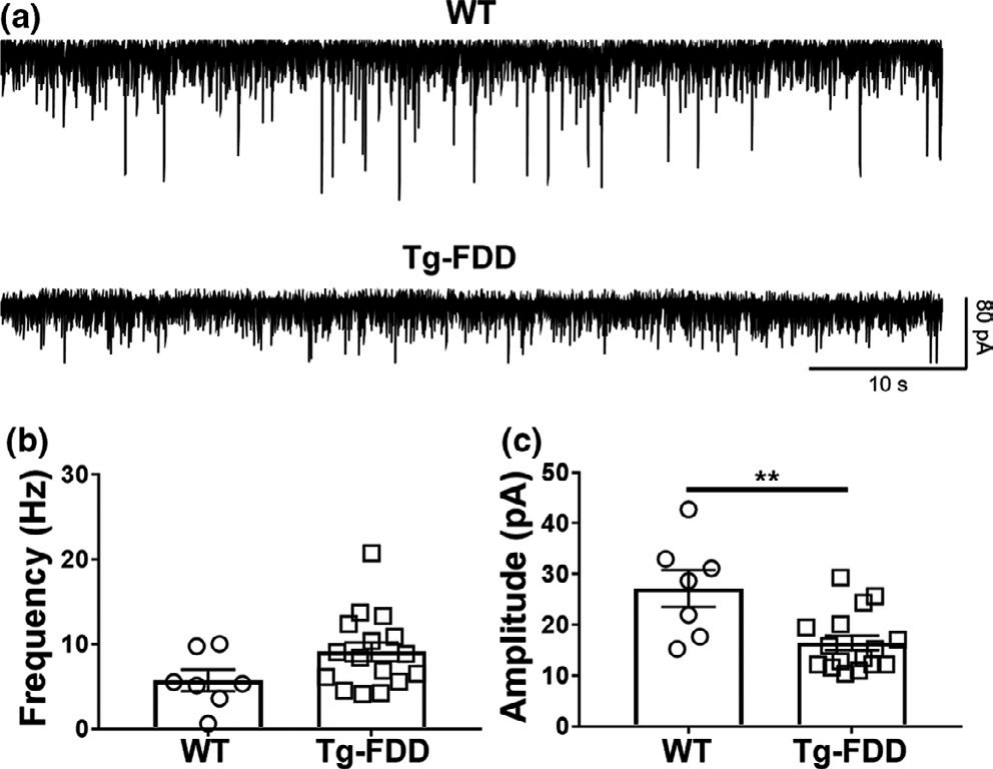
Spontaneous inhibitory transmission is affected in CA1 hippocampal neurons from Tg‐FDD mice. (A) Representative synaptic traces in the presence of NBQX (10 µM) and D‐APV (50 µM), recorded at a −60 mV holding potential in CA1 hippocampal neurons from WT and Tg‐FDD mice. (B) No differences were found in the frequency of the sIPSCs between WT and Tg‐FDD mice. (C) The amplitudes of sIPSCs were reduced in brain slices from Tg‐FDD mice (*p* = 0.003, t21 = 3.348, unpaired Student's *t* test; *n* = 7 neurons from 2 WT mice, *n* = 16 neurons from three Tg‐FDD mice). Data represented as mean ±SEM.

Altered behavior is strongly associated with synaptic damage (Lepeta et al., 2016). Therefore, we examined the function and organization of synapses in Tg‐FDD mice to determine whether synaptic impairment correlates with the observed hyperactive behavior. Hippocampal long‐term potentiation (LTP) experiments were carried out on the CA1 hippocampal area of 18‐month‐old WT and Tg‐FDD mouse‐derived brain slice preparations. We monitored field excitatory postsynaptic potentials (fEPSPs) evoked by extracellular stimulation of the Schaffer collateral pathway and induced LTP using high‐frequency stimulation (four stimuli of 100 Hz for 1 s with a 10‐s inter‐stimulus interval). No differences in fEPSPs were observed between Tg‐FDD and WT mice (Figure S1). We then assessed GABA transmission by measuring spontaneous inhibitory postsynaptic currents (sIPSCs) recordings in CA1 pyramidal cells within coronal brain slices containing the hippocampus in a second cohort of 18‐month‐old WT and Tg‐FDD mice. These analyses revealed a significant decrease in the amplitude sIPSCs in brain slices from Tg‐FDD mice in comparison with WT, with no change in sIPSC frequency (Figure 2). This synaptic functional analysis suggests an impairment of inhibitory synapses in the Tg‐FDD model.

**FIGURE 3:**
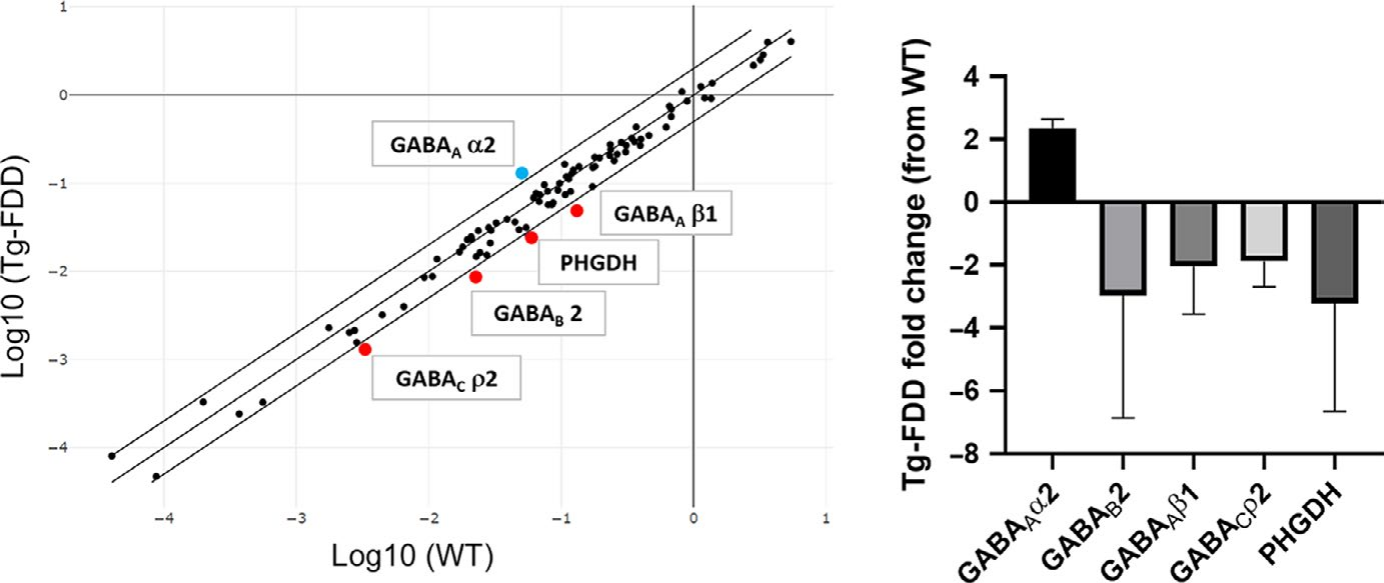
Dysregulation of inhibitory synapse‐related genes in Tg‐FDD mice. (A) Gene plot of Tg‐FDD versus WT of 84 genes of both excitatory and inhibitory synapse pathways. Red and blue dots correspond to genes down‐ and upregulated in Tg‐FDD, respectively. (B) Fold change plot of genes down‐ and upregulated in Tg‐FDD. *p* < 0.05, unpaired Student's *t* test, fold change threshold = 2, *n* = 3 different brains for each genotype. Data represented as mean ±SEM.

We then performed quantitative PCR to determine the expression level of 84 gene markers from both excitatory and inhibitory synapses in brain tissue from 18‐month‐old Tg‐FDD and WT mice (Figure 3A and B). Only the inhibitory synapse components were dysregulated in Tg‐FDD compared with WT mice (Figure 3A and B). The synaptic components *GABA_A_Rβ1* (fold change = −2.62), *GABA_B_2* (fold change = −2.66), *GABAρ2* (fold change = −2.44), and phosphoglycerate dehydrogenase (*PHGDH*, fold change = −2.2) were downregulated in Tg‐FDD mice. *GABA_A_Rα2* was upregulated (fold change = 2.35). Interestingly, no excitatory synaptic components were down‐ or upregulated between Tg‐FDD mice and WT. These results further indicate inhibitory synaptic impairment in the Tg‐FDD model.

**FIGURE 4:**
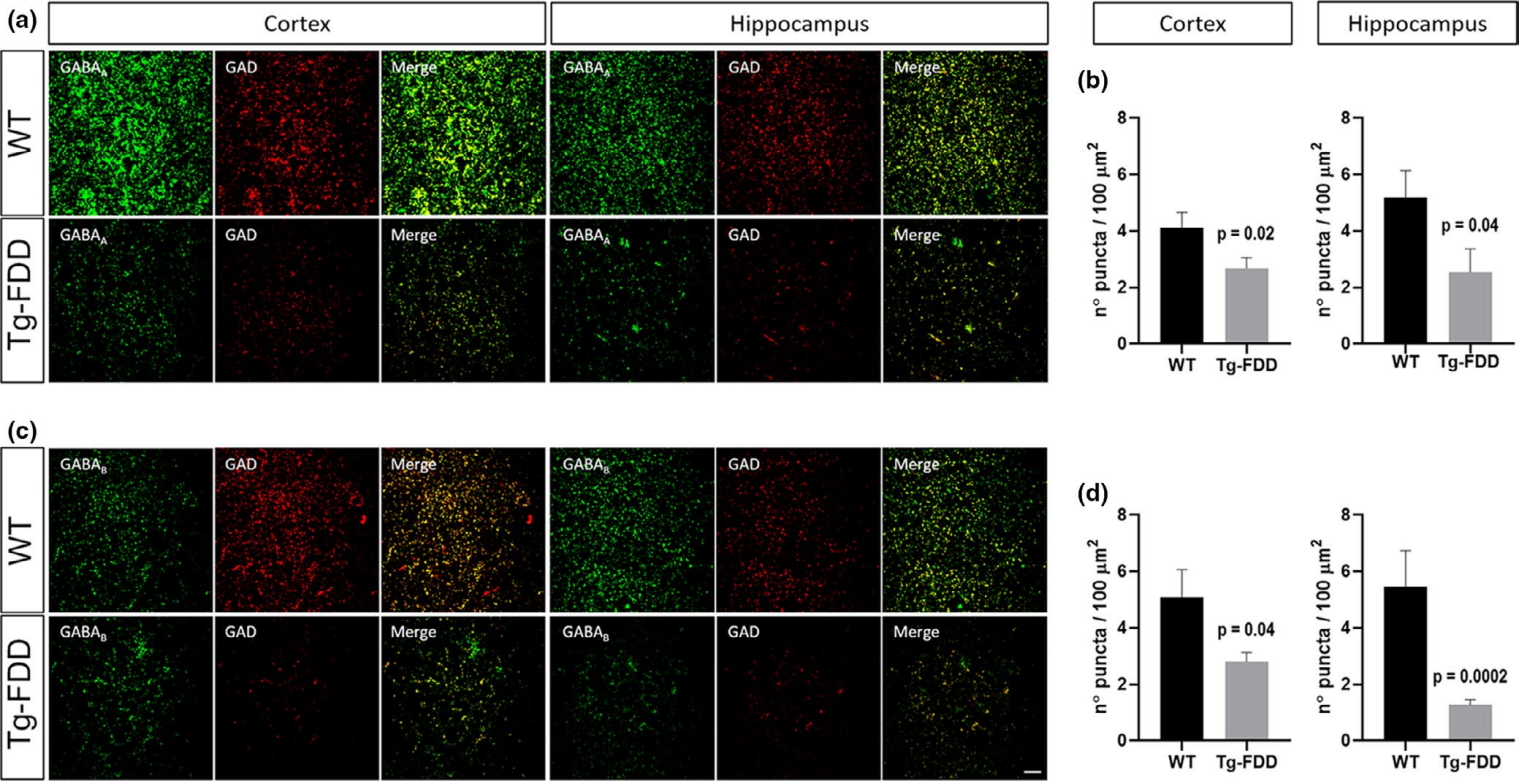
Decrease in the synaptic localization of inhibitory synaptic markers in Tg‐FDD mice. (A) Immunofluorescence for GABA_A_ receptor (green) and GAD (red) on cortex and hippocampus. (B) Quantification of the number of merge puncta for both markers on A. (C) Double staining for GABA_B_ receptor (green) and GAD (red). (D) Quantification of the number of merged puncta for both markers on C. *p* < 0.05 indicated on each graph, Mann–Whitney test, *n* = 9 photographs from 3 different animals per genotype. Data represented as mean ± SEM. Scale bar 10 μm.

**FIGURE 5:**
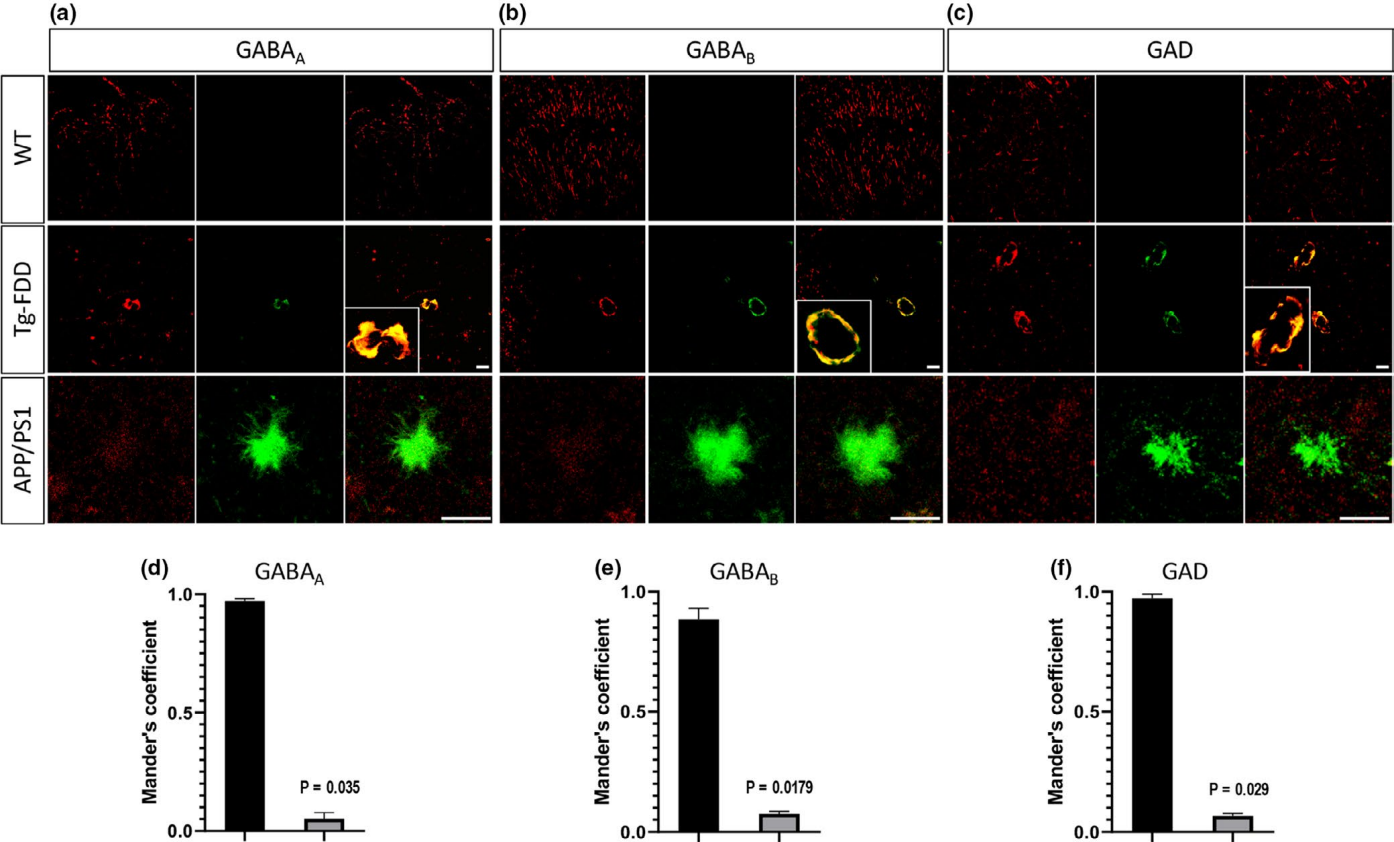
Inhibitory synaptic markers co‐deposit with vascular amyloid in Tg‐FDD mice. (A–C) Double immunofluorescences for GABA_A_, GABA_B_ receptors or GAD (red), and Thio‐S (green) in brain sections of Tg‐FDD, APP/PS1, and WT mice. Thio‐S colocalizes with all three markers in Tg‐FDD mice as shown in merge and inset. (D–F) Mander's coefficients for Thio‐S and inhibitory synaptic marker colocalization were calculated from selected images. *p* < 0.05 indicated on each graph, Mann–Whitney test, *n* = 9 photographs from 3 different animals per genotype. Data represented as mean ± SEM. Scale bars 30 μm in WT and Tg‐FDD and 20 μm in APP/PS1.

To determine any disruption in the organization of excitatory and inhibitory synapses, we performed immunofluorescence analysis in brain sections from Tg‐FDD and WT mice. To measure excitatory synapse organization, we quantified synaptic puncta positive for two excitatory markers, synapsin‐1 and GluN1 (Cisternas et al., 2016). No changes were observed in the number of colocalized synapsin‐1 with GluN1 puncta between Tg‐FDD and WT brains, suggesting that the organization of excitatory synapses is not impaired in this model for CAA (Figure S2). Inhibitory synapse organization was determined by quantifying positive (+) puncta for the inhibitory synapse markers GABA_A_R or GABA_B_R and GAD65/67. In Tg‐FDD brains, the number of synaptic clusters of both GABA receptors in relation to GAD65/67 was significantly lower than in WT mice (Figure 4), indicating a disruption in the organization of inhibitory synapses. All quantifications to establish excitatory and inhibitory synapse organization (Figure S2 and Figure 4) were performed in areas of the cortex and hippocampus where no major vascular amyloid deposition was observed. Interestingly, in certain areas of the hippocampus and cortex, we observed major deposits of inhibitory synapse markers (data not shown). Therefore, we performed double immunofluorescence for inhibitory synapse markers and thioflavin‐S on the Tg‐FDD mice that accumulate ADan amyloid in the vasculature to establish whether these deposits of inhibitory synapse markers localized with vascular amyloid deposits. Surprisingly, we observed a strong colocalization between vascular amyloid signal and all three GABA markers. This colocalization was specific to vascular amyloid, since no association between GABA_A,_ GABA_B_ receptors, and GAD65/67 with parenchymal amyloid plaques was observed in an APP/PS1 mouse model (Figure 5A–C). The quantification of the colocalization between amyloid and these inhibitory synaptic markers confirmed our findings (Figure 5D–F). Overall, these results suggest that the hyperactive behavior observed in Tg‐FDD mice may result from functional impairment of the inhibitory synapses due to disruption in their organization.

### 2.3 Alteration of inhibitory synapse in CAA patients

**FIGURE 6:**
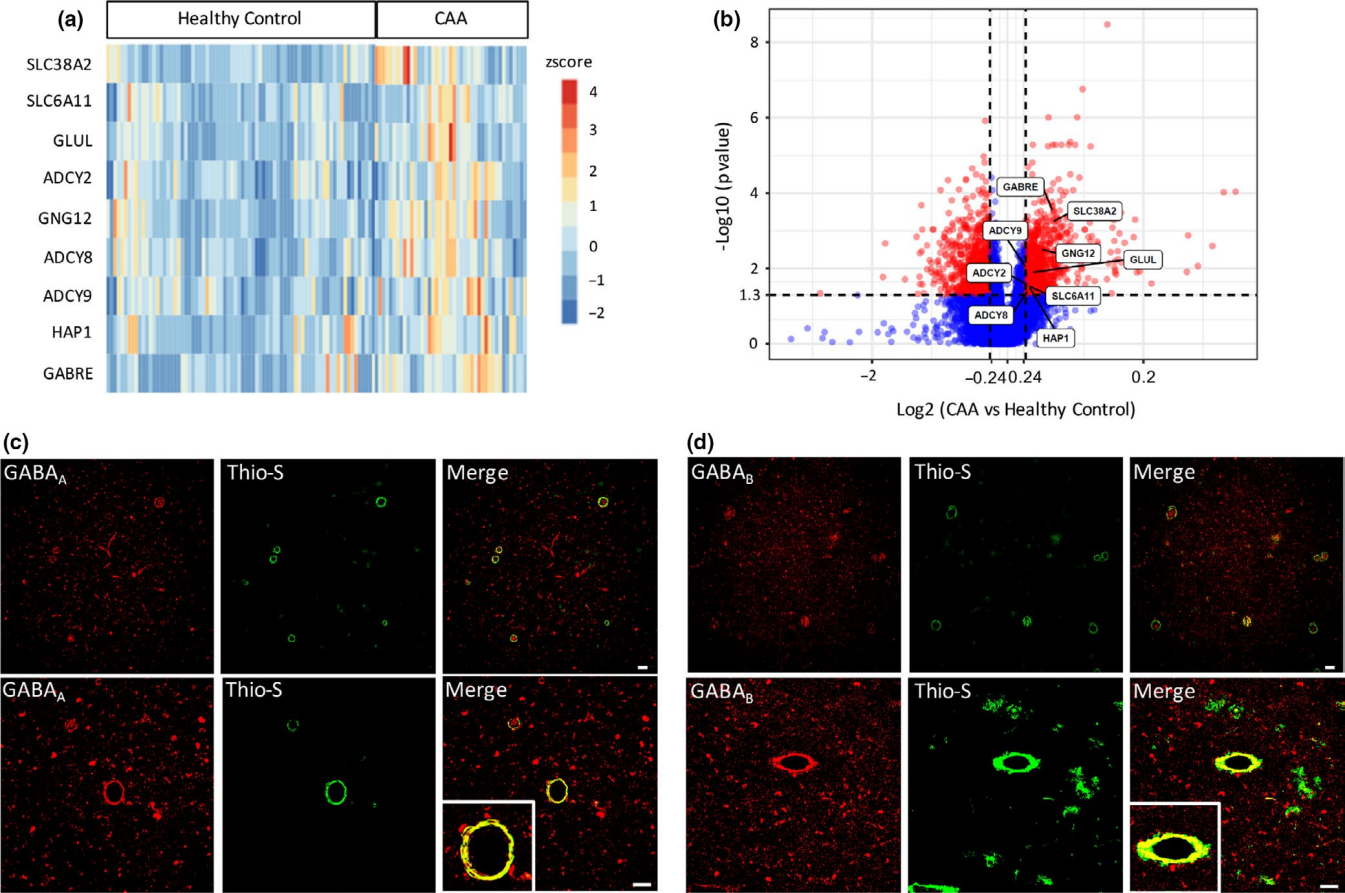
GABAergic synapse signature in human cases with CAA pathology. (A, B) Heatmap and volcano plot comparing significantly changed genes associated with the GABAergic pathway in patients with CAA pathology and age‐matched controls identified by available RNA‐Seq data sets. (C, D) Co‐deposition of GABA_A_ and GABA_B_ receptors (red) and vascular amyloid deposits detected with Thio‐S (green) in a patient with CAA pathology. Scale bar 60 μm.

To establish whether similar synaptic alterations are observed in patients with CAA pathology, we analyzed RNA‐Seq data from healthy human controls and CAA pathology brain samples from the AMP‐AD Knowledge Portal (Allen et al., 2016). These analyses revealed that the inhibitory GABAergic synapse pathway genes are highly enriched in CAA brain samples compared with healthy controls (Figure S3A). Specifically, adenylate cyclase 2, 8, and 9 (*ADCY2*, *ADCY8*, and *ADCY9*), the solute carrier family 6 member 11, family 38 member 2, family 1 member 3, and family 17 member 8 transporters (*SLC6A11*, *SLC38A2*, *SLC1A3*, and *SLC17A8*), the Huntingtin‐associated protein 1 (*HAP1*), the G‐protein subunit γ 12 (*GNG12*), the glutamate‐ammonia ligase (*GLUL*), the metabotropic glutamate receptor 1 (*GRM1*), and GABA_A_R subunit ε (*GABRE*) (Figure 6A,B and Figure S3B). Finally, we performed double immunofluorescence for GABA receptors and amyloid in a previously characterized A*β*–CAA case (Vidal et al., 2000). As in the Tg‐FDD model (Figure 5A–C), a strong colocalization between vascular amyloid signal and GABA synaptic markers was observed (Figure 6C). We found colocalization with both GABA_A_ and GABA_B_ receptors in all twenty‐five cerebral vessels with amyloid deposits. Overall, these results suggest disrupted inhibitory synapse organization and related functional impairment observed in the Tg‐FDD mice may also represent a pathological phenotype in patients with CAA pathology.

## 3 DISCUSSION

Our data indicate that aged Tg‐FDD mice, a model for CAA, exhibit a hyperactive phenotype accompanied by dysfunction of inhibitory synapses, as demonstrated by a reduction of sIPSCs and a disruption in the organization of GABAergic markers. Interestingly, these markers were co‐deposited with vascular amyloid. Histological analysis of brain samples from patients with CAA pathology complemented these findings, in which we observed dysregulation of inhibitory synapse‐related pathways and an accumulation of GABAergic synapse markers in the vasculature.

Vascular amyloid accumulation is identified in an estimated 70%–97% of individuals with AD (Attems, 2005). Despite this strong co‐occurrence, the synaptic damage present in AD and its correlation with cognitive and behavioral alterations has mainly been studied in the context of parenchymal amyloid accumulation (Rudy et al., 2015). The contribution of vascular amyloid to this matter has been understudied, although CAA is considered one of the strongest vascular contributors to age‐related cognitive decline (Attems, 2005; Holton et al., 2002; Vinters & Gilbert, 1983). Herein, we demonstrated in a mouse model for CAA without major parenchymal amyloid how vascular amyloid accumulation could be responsible for a hyperactive phenotype, possibly due to alterations in GABAergic synapses. Interestingly, a hyperactive phenotype has been previously reported in two different transgenic mouse models for AD characterized by the overexpression of mutant human APP and major deposits of parenchymal amyloid plaques (Filali, Lalonde, & Rivest, 2011; Roberson et al., 2007). It is noteworthy that vascular amyloid has been observed in both models (Badhwar, Brown, Stanimirovic, Haqqani, & Hamel, 2017; Kelly et al., 2017), indicating that vascular pathology may also contribute to a hyperactive phenotype in these models.

Mechanistically, an in vivo study using the APP23xPS45 mouse model has shown that an increase in hyperactive clusters of neurons in the vicinity of Aβ plaques resulted from a decrease in synaptic inhibition as opposed to enhancement of excitatory synaptic transmission (Busche et al., 2008). Similarly, β‐amyloid has been reported to disrupt the inhibitory input on neurons of the cingulate cortex (Ren et al., 2018), and inhibitory neurons are the target of neurodegeneration in the TauPS2APP mouse model of amyloidosis and tauopathy (Loreth et al., 2012). The hypothesis that hyperactive behavior observed in different models of amyloidosis is due to an alteration in GABAergic synapses is supported by the fact that mice lacking the GABA transporter subtype 1 (GAT1), responsible for the reuptake of GABA after its release, exhibit a hyperactive phenotype (Chen et al., 2015), and that mice lacking GAD67, the enzyme that catalyzes the decarboxylation of glutamate to synthesize GABA, also show a hyperactive phenotype (Smith, 2018).

In our study, we show a decrease in the amplitude of sIPSCs but no change in fEPSPs. Interestingly, GABAergic input is an important component of LTP and modulation of memory and learning, contributing to its inhibitory control (Steele & Mauk, 1999). Nevertheless, the decrease in GABAergic input does not necessarily indicate an impairment in the glutamatergic input, which is the main component of LTP. Therefore, LTP as a consequence of unaffected fEPSPs observed in the Tg‐FDD model may result from spontaneous glutamatergic neuronal activity.

As the GABAergic synapse pathway is highly enriched in upregulated genes in human CAA versus healthy control brain samples, the inhibitory synaptic impairment observed in the Tg‐FDD mice may also be present in patients with CAA pathology. Nevertheless, the patients from which the CAA brain samples were derived were also diagnosed with AD; therefore, it is not possible to rule out the contribution of parenchymal amyloid in the dysregulation of GABAergic synapse pathway, although we did not find co‐deposits of GABAergic synapse markers with parenchymal amyloid in the APP/PS1 mouse model. Considering the synaptic characterization of the Tg‐FDD model in conjunction with the available RNA‐Seq from patients with CAA, synaptic impairment may result from a disruption in the organization of GABAergic components in addition to an overall dysregulation of inhibitory synaptic genes.

Our immunofluorescence analysis demonstrated that GABA receptors co‐deposit with vascular amyloid in the Tg‐FDD model as well as a human case for CAA, supporting the hypothesis that that inhibitory synapse impairment results from a loss of synaptic organization of GABA receptors. In support of this association, novel quantitative proteomic analysis of vascular amyloid confirmed a differential expression in several GABAergic proteins in leptomeningeal arteries from CAA patients versus age‐matched controls (Manousopoulou et al., 2017). The colocalization of GABA receptors with ADan and Aβ vascular amyloid supports the idea that CAA phenotypes are driven by the accumulation of amyloid deposits in the walls of arterial blood vessels, independent of the origin of the proteins (Cisternas et al., 2019). The notion of a unique CAA phenotype is supported by major neuropathological resemblances between the Tg‐FDD model and Aβ‐CAA. For instance, accumulation of ADan amyloid in the Tg‐FDD is accompanied by a loss of vascular smooth muscle cells (Vidal et al., 2009), as described in Aβ‐CAA (Attems, 2005). Other cellular and pathological similarities between the Tg‐FDD model and Aβ‐CAA include amyloid‐associated gliosis, a strong inflammatory response, association of ApoE with vascular amyloid deposits, and the presence of perivascular tau aggregates (Vidal et al., 2009; You et al., 2019).

The molecular mechanism underlying amyloid‐related disruption of the synaptic position of GABA receptors is not well understood. It has been reported that Aβ can interact with the GABA_A_R, promoting its endocytosis (Ulrich, 2015); however, there are no studies addressing this matter in relation to vascular amyloid. CAA‐related brain injuries arise from blood vessel dysfunction (Greenberg et al., 2014), either via loss of vessel integrity, as previously reported in the Tg‐FDD model (Vidal et al., 2009), or via loss of normal blood supply and ischemia. Therefore, vascular amyloid may indirectly promote GABAergic impairment by promoting the loss of blood–brain barrier integrity and subsequently triggering a series of molecular events that directly affect inhibitory synapse function. This indirect mechanism is supported by a previous study demonstrating GABA alteration and GABA_A_R involvement in blood–brain barrier disruption in cerebral ischemia (Chi, Hunter, Liu, Chi, & Weiss, 2011). Interestingly, a novel study has demonstrated that GABA_A_R modulates the plasma membrane potential in arterial vascular smooth muscle cells (Wang, Cheng, & Schmid, 2015). As vascular amyloid triggers an impairment of vascular smooth muscle cells in CAA (Attems, 2005), GABA_A_R dysfunction in these vascular cells may contribute to the cerebrovascular pathology observed in CAA.

It has been theorized that soluble amyloid oligomers exert neurotoxicity as opposed to amyloid plaques via a diverse set of molecular pathways (Kayed & Lasagna‐Reeves, 2013) including GABAergic synapse dysfunction (Krantic et al., 2012). As ADan oligomers localize intracellularly and in the vicinity of vascular amyloid deposits in 18‐month‐old Tg‐FDD mice (You et al., 2019), the possibility that ADan oligomers are responsible for the GABAergic impairment that we observed in this model for CAA is an additional potential mechanism of synaptic damage.

Hyperphosphorylated forms of tau are also present in the Tg‐FDD model and necessary for ADan oligomers to exert neurotoxicity, suggesting a preponderant role of tau in the CAA‐related neurodegeneration (You et al., 2019). Several studies have demonstrated that tau aggregation promotes GABAergic dysfunction in vivo (Levenga et al., 2013). Reducing tau levels rescues the abnormal hyperactive phenotype observed in an hAPP mouse model characterized by the accumulation of parenchymal amyloid plaques (Roberson et al., 2007). This reduction of tau confers protection against the lethal effects of GABA_A_ receptor antagonist in this model (Roberson et al., 2007), suggesting that the protective role of tau reduction occurs via a GABAergic‐mediated mechanism. Based on these findings and the established relationship between tau and the Tg‐FDD model, the role of tau in the Tg‐FDD model may also be involved in the hyperactive phenotype associated with GABAergic dysfunction.

In conclusion, this study demonstrates the contribution of vascular amyloid deposition to GABAergic dysfunction and hyperactive phenotype in the Tg‐FDD mouse model and that the GABAergic pathway also is impaired in comparison with healthy controls in human patients with CAA pathology. Vascular amyloid pathology likely plays a significant role in synaptic impairment, highlighting the importance of characterizing the mechanism by which CAA contributes to synaptic dysfunction in AD‐related dementias and age‐related cognitive decline.

## 4 EXPERIMENTAL PROCEDURES

### 4.1 Transgenic mouse model

Tg‐FDD male and female mice were used for our experiments. These mice express a FDD‐associated human mutant BRI2 transgene that leads to the vascular accumulation of the ADan amyloid (Vidal et al., 2009). Mice were housed at the Indiana University School of Medicine (IUSM) animal care facility and were maintained according to USDA standards (12‐h light/dark cycle, food and water ad libitum), in accordance with the Guide for the Care and Use of Laboratory Animals (National Institutes of Health, Bethesda, MD). Animals were anesthetized and euthanized according to IUSM Institutional Animal Care and Use Committee‐approved procedures. For all described experiments, 18‐month‐old animals were utilized. For immunofluorescence, mice were perfused transcardially with PBS prior to decapitation. Brains were extracted and formalin‐fixed for the preparation of paraffin blocks as previously described (Vidal et al., 2009).

### 4.2 Behavioral tests

All animals used in behavioral tests were 18‐month‐old WT C57BL/6 and Tg‐FDD mice. All experiments were carried out and analyzed with the experimenter blind to the treatment group.

*Spontaneous alternation Y*‐*Maze*: To assess sustained cognition and spatial learning, mice were subjected to the Y‐Maze test as described in (Miedel, Patton, Miedel, Miedel, & Levenson, 2017). Mice were allowed to freely explore the maze for a period of 10 min. An arm entry was defined when all four paws of the mouse crossed a linear threshold determined by the nose, and the mouse snout is oriented toward the arm. A spontaneous alternation occurs when a mouse enters a different arm of the Y‐Maze in each of three consecutive arm entries. Each session was recorded, and data acquisition was manually acquired. Spontaneous alternation percentage was calculated as ((# spontaneous alternation)/(total number of arm entries − 2)) × 100.

*Novel object recognition*: To further assess cognitive processing, mice were subjected to the novel object recognition (NOR) test as described in Miedel et al. (2017). On the first day, mice were habituated to the NOR recognition box for 10 min and returned to their cages. The next day, mice were habituated for an additional 2 min, and then, two identical objects were placed in the box. In this training session, mice were given 10 min to explore and then were returned to their cages. 24 h later, mice were returned to the box where one of the objects was replaced with a completely different object in color, size, and texture. They were allowed to explore for 10 min. All sessions were recorded. Time spent interacting with each object was manually analyzed.

*Rotarod*: To assess locomotor function and activity, mice were tested on a rotarod device (Columbus Instruments International) for 4 days in a row as described previously (Ure et al., 2016). Each day had four trials; each trial lasted a maximum of 10 min, during which the rotating rod underwent a linear acceleration from 4 to 40 rpm over the first 5 min of the trial and then remained at maximum speed for the remaining 5 min. Animals were scored for their latency to fall (in s) in each trial. Animals rested a minimum of 30 min between trials to avoid fatigue and exhaustion.

*Cylinder test*: To further assess spontaneous activity, mice were subjected to the Cylinder test as described in Fleming et al. (2013). Briefly, mice were placed in a transparent cylinder with an angled mirror that allowed the recording of the time spent in a vertical position with both forelimbs off the floor so that the mouse is standing only on its hindlimbs. Each mouse was manually recorded for a period of 3 min.

*Open*‐*Field test*: Mice were tested with the open‐field test, as described in Ure et al. (2016). Briefly, mice were placed in the center of a dimly lit (20–30 lux) open‐field arena (61 × 61 × 61 cm) for 10 min, allowing them to freely explore the arena. Movements of the animals were tracked by an automatic monitoring system (ANY‐maze) for 10 min. The area was virtually divided into three types of quadrants: a center (40 cm edge lengths), four wall corridors (7.5 cm along the walls), and four corner squares (with 10 cm edge lengths). Time was automatically measured for each quadrant.

### 4.3 Electrophysiology

*Brain slice preparation*: Immediately following euthanasia via decapitation under deep isoflurane anesthesia, the brain was quickly excised and placed in an ice‐cold tissue cutting solution containing (in mM): 194 sucrose, 30 NaCl, 4.5 KCl, 1 MgCl_2_, 26 NaHCO_3_, 1.2 NaH_2_PO_4_, 10 glucose saturated with a mixture of 95% O2 and 5% CO2, and coronally sliced to a thickness of 280 μm on a vibratome (Leica VT1200S) for whole‐cell recordings, and sagittal cuts of 350 μm thickness for field recordings. Slices were transferred to an artificial cerebrospinal fluid (aCSF) solution containing (in mM): 124 NaCl, 4.5 KCl, 1 MgCl_2_, 26 NaHCO_3_, 1.2 NaH_2_PO_4_, 10 glucose, 2 CaCl_2_ (310–320 mOsm) saturated with 95% O2/5% CO2 at 30°C for 1 h before being moved to room temperature (RT). When ready for recording, slices were transferred to a recording chamber continuously perfused with aCSF solution saturated with 95% O2/5% CO2.

*Field potential recordings*: Field excitatory postsynaptic potential (fEPSP) recordings were made from the stratum radiatum of hippocampal area CA1 using a MultiClamp 700B amplifier (Axon Instruments). Signals were amplified (gain 100) and filtered (1 kHz), then digitized (10 kHz). Slices were visualized on an Olympus BX51WI microscope (Olympus Corporation of America). Experiments were carried out at 29–32°C, and aCSF was continuously perfused over the brain slices at a rate of 1–2 ml/min Picrotoxin (50 μM) was added to the aCSF for recordings to isolate excitatory potentials. Micropipettes were prepared from filament‐containing borosilicate micropipettes (World Precision Instruments) using a P‐1000 micropipette puller (Sutter Instruments), having a 2.0–3.5 MΩ resistance. The glass micropipettes were filled with 1 M NaCl and placed into the stratum radiatum of hippocampal area CA1. A concentric bipolar stimulating electrode (FHC) was placed into stratum radiatum in area CA1 ∼500 μm from the recording site. fEPSPs were generated by a DS3 Isolated Current Stimulator (Digitimer) every 20 s, and stimulus intensity was adjusted to produce stable fEPSP responses prior to the initiation of experimental recording. A 10‐min baseline was recorded before delivery of four high‐frequency stimulations (HFS; 100 Hz, 1‐s duration, 10‐s inter‐stimulus‐interval). Data were acquired using Clampex 10.3 (Molecular Devices). The representative traces were obtained from the average baseline fEPSP (1–10 min) and average post‐HFS fEPSP of final 10 min of recording. Exclusion of individual data points was determined using the Grubb's test, an outlier calculator included in the Prism 7 software package. Recordings were made 2–7 h after euthanasia.

*Whole*‐*cell recordings*: Coronal brain slices containing the hippocampus were prepared from adult WT and Tg‐FDD, as described above. Whole‐cell recordings of spontaneous inhibitory postsynaptic currents (sIPSCs) in CA1 hippocampal pyramidal neurons were carried out at 29–32°C, and aCSF was continuously perfused at a rate of 1–2 ml/min. Slices were visualized on an Olympus BX51WI microscope (Olympus Corporation of America). Patch pipettes were prepared from filament‐containing borosilicate micropipettes (World Precision Instruments) using a P‐1000 micropipette puller (Sutter Instruments), having a 2.0–3.5 MΩ resistance. The internal solution contained (in mM): 120 CsCl, 4 MgCl_2_, 10 HEPES, 5 lidocaine bromide, 10 EGTA, 0.5 Na‐GTP, and 2 Mg‐ATP (pH 7.2–7.4 and 290–310 mOsm). Pyramidal neurons were held at a −60‐mV holding potential throughout the recordings. We pharmacologically isolated the inhibitory spontaneous synaptic activity via bath application of the AMPA receptor antagonist, 2,3‐dioxo‐6‐nitro‐1,2,3,4‐tetrahydrobenzo[f]quinoxaline‐7‐sulfonamide (NBQX, 10 µM), and NMDA receptor antagonist, D‐2‐amino‐5‐phosphonovalerate (D‐APV, 50 µM). Recordings were performed using a MultiClamp 700B amplifier and a Digidata 1550B (Axon Instruments). Currents were displayed and stored on a personal computer using Clampex 10.3 (Molecular Devices) and analyzed with MiniAnalysis 6.0 (Synaptosoft Inc.). Series resistance was monitored, and only cells with a stable series resistance (<25 MΩ and that did not change more than 15% during recording) were included for data analysis. Recordings were made 2–7 h after euthanasia.

### 4.4 Quantitative PCR

Analysis of 84 different excitatory and inhibitory synapse gene markers was analyzed with the RT^2^ Profiler PCR Array for mouse GABA and glutamate synaptic pathways (330231 PAMM‐152ZA, Qiagen). Briefly, total RNA from mouse brains was isolated with RNeasy Plus Universal Mini Kit (Qiagen). Genomic DNA elimination and cDNA were done incubating 0.5 μg of RNA with the RT^2^ First Strand Kit (330401, Qiagen). Relative gene expression was evaluated with the delta Ct method. Data analysis was performed using the Qiagen Data Analysis Center (https://geneglobe.qiagen.com/us/analyze/).

### 4.5 Brain section immunofluorescence (IF)

Paraffin sections from the brains of 18‐month‐old WT, Tg‐FDD, and APP/PS1 (Jankowsky et al., 2004) animals were deparaffinized in xylene, rehydrated in ethanol (EtOH), and washed with deionized water. Then, the sections were heated twice in a low pH antigen retrieval solution (eBioscience) for 4 min each. Sections were blocked with PBS 5% goat 5% horse serum 2% fish gel 0.01% Triton X‐100 for 1 h at RT and then incubated overnight at 4°C with a 1/100 solution of the following antibodies: GABA_A_Rα1‐6 (sc‐376282, Santa Cruz), GABA_B_R1 (MA5‐27704, Thermo Fisher), GAD65/67 (PA1‐84572, Thermo Fisher), Synapsin‐1 (ab64581, Abcam), and GluN1 (NMDAR1 556308, BD Pharmingen). The next day, sections were quickly washed three times in PBS and incubated with 1/500 biotinylated horse (BA‐200, Vector) and/or goat (BA‐1000, Vector) secondary antibodies for 1 h at RT. 30 min in advance, the Vectastain Elite ABC peroxidase kit (PK‐6100, Vector) was prepared according to the manufacturer's instructions. After the secondary antibody incubation, sections were incubated with the A + B solution for 30 min at RT. After three quick washes with PBS, tyramide dyes were prepared 1/500 in PBS and slides were incubated with them for 10 min at RT. To stop peroxidase activity in case of performing a double staining, slides were incubated with 3% H_2_O_2_ for 10 min at RT. After, they were quickly washed with PBS. To stain vascular amyloid, 1% thioflavin‐S was used for 8 min at RT, followed by two washes in EtOH 30% and 50% for 3 min each. A final wash in deionized water for 5 min was followed by the mounting procedure with Vectashield mounting media. Quantification of the colocalization of synaptic markers with amyloid and determination of the Mander's coefficient were performed using the ImageJ program as previously described (Sharma, Zhang, & Huang, 2018).

### 4.6 Human data source, pre‐processing, and expression analysis.

RNA‐Seq data of human CAA and AD brain samples were obtained from the Accelerating Medicines Partnership‐Alzheimer's Disease (AMP‐AD) Knowledge Portal (MAYO TCX synapse ID: syn8612203, MAYO MC‐CAA: syn9779506). MAYO TCX cohort samples were collected from the temporal cortex region of AD and healthy control brains (Allen et al., 2016), while MAYO MC‐CAA cohort was generated from the temporal cortex region of AD brains, some of which are CAA. There are 75 AD samples in MAYO MC‐CAA, in which 43 are CAA and 32 are non‐CAA. In MAYO TCX cohort, there are 82 AD samples and 78 healthy control samples. We pre‐processed the raw gene read counts of both cohort samples separately. First, we discarded genes with 50% samples of zeros. Then, we removed genes with maximum read counts less than 20. Finally, for multiple transcripts with the same gene name, we only retained the transcript with the highest expression value to represent the gene. After filtering, we compared the genes of two filtered cohort samples and obtained 21,213 genes that occurred in both cohorts for further analysis. Processed raw read counts of both cohorts were normalized by DEseq2, ComBat, which is included in the sva package (Leek, Johnson, Parker, Jaffe, & Storey, 2012), and was used on the log‐transformed normalized read counts to remove the batch effect of the two data sets. After normalization and adjusting for batch effect, *t* test was used for the differential expression analysis between CAA and healthy control samples with the cutoff of fold change > 1.2, FDR‐adjusted *p*‐value < 0.05.

### 4.7 CAA patient case

Immunofluorescent analysis was performed in a paraffin section of a single case of amyloid angiopathy previously described (Vidal et al., 2000), where the subject diagnosed with senile dementia was homozygous for ApoE4 without mutations in APP and PS1 genes.

### 4.8 Statistical analysis

Experimental analysis and data collection were performed in a blinded fashion, if not stated otherwise. *P*‐values were calculated using Student's unpaired *t* test and the non‐parametric Mann–Whitney test, whenever distributions were not normal in the GraphPad Prism software. Significance was assigned to *p* < 0.05. All details of experiments can be found in the figure legends.

## CONFLICT OF INTEREST

No conflict of interest to declare.

## AUTHORS CONTRIBUTIONS

CAL‐R and PC conceived and coordinated the study. PC, EA, and YM assisted in animal maintenance and breeding. PC, XT, AP, and EA performed behavioral studies. PC, OM, and RC performed immunohistochemistry and quantification. PC and XT performed qPCR. BA, BM, and TP performed and coordinated electrophysiology experiments. RV provided Tg‐FDD mice and human CAA cases for histology. JZ and SX performed and coordinated gene expression analysis from RNA‐Seq available data sets. CAL‐R, PC, BA, BM, SX, and JZ performed analysis of data and drafted the images for publication. PC, CAL‐R, AP, and BM wrote the manuscript. All authors read and approved the final manuscript.
