# Supplementary figures and images for: Vascular amyloid accumulation alters the gabaergic synapse and induces hyperactivity in a model of cerebral amyloid angiopathy
Source: Aging Cell. 2020 Sep 10;19(10):e13233. doi: 10.1111/acel.13233 (PMC7576303; doi:10.1111/acel.13233)

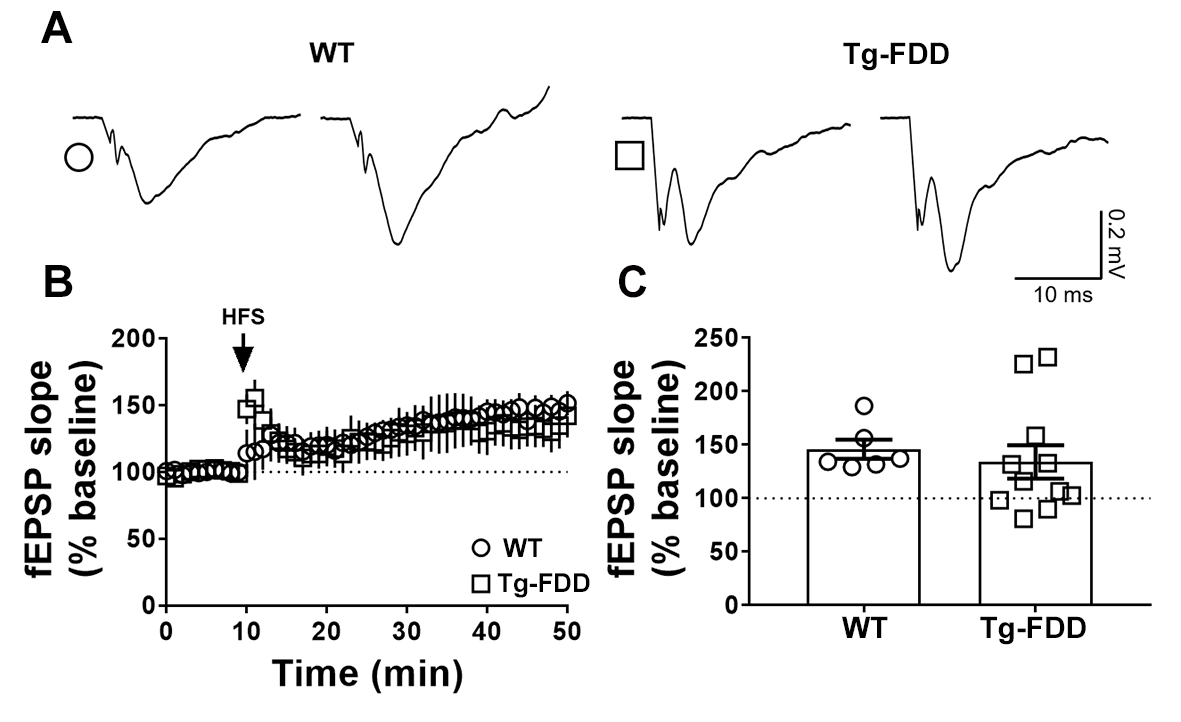

Supplement: Supplementary file 1 [file ACEL-19-e13233-s001.tif]

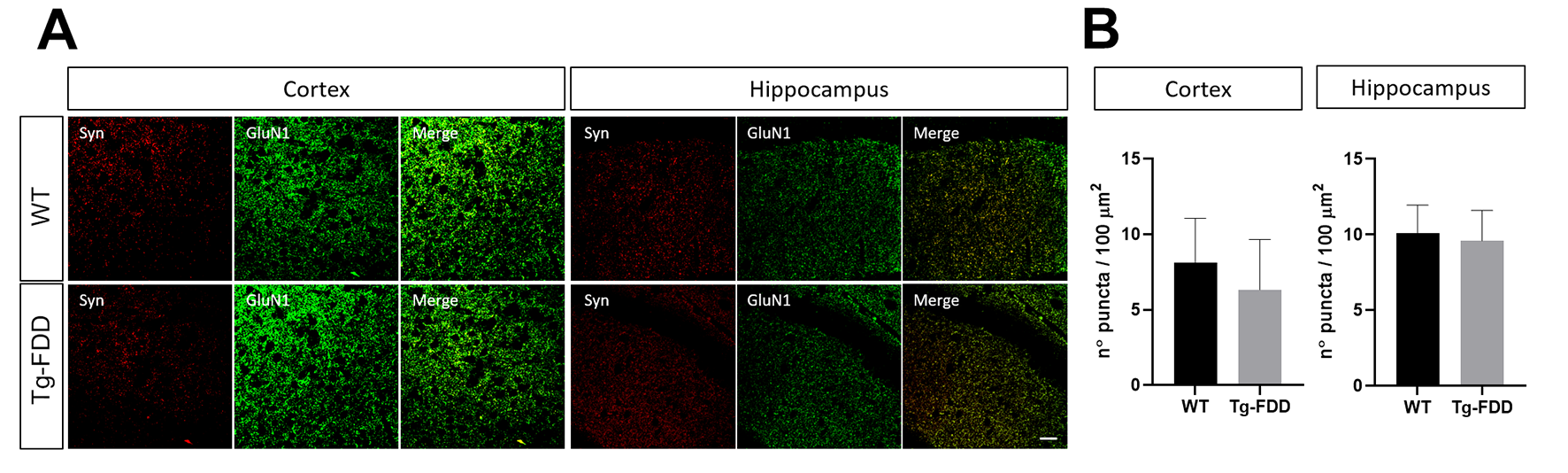

Supplement: Supplementary file 2 [file ACEL-19-e13233-s002.tif]

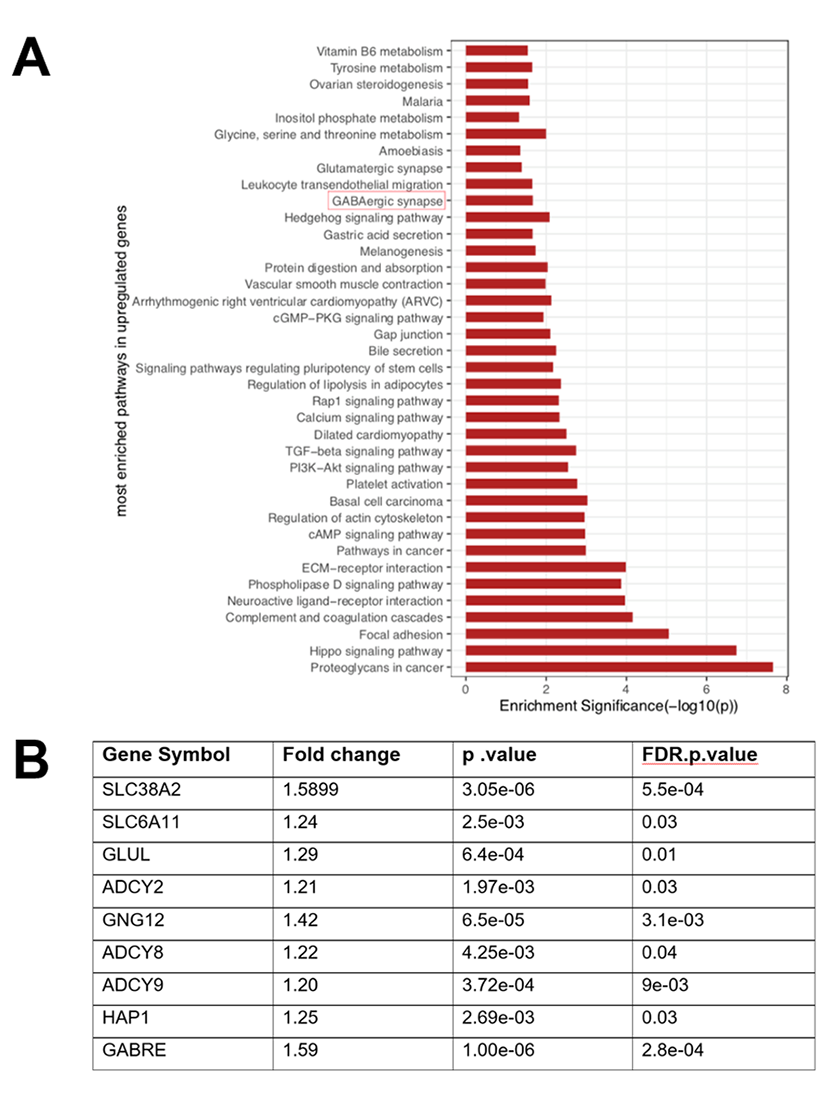

Supplement: Supplementary file 3 [file ACEL-19-e13233-s003.tif]
