# Supplementary material for: Vascular amyloid accumulation alters the gabaergic synapse and induces hyperactivity in a model of cerebral amyloid angiopathy
Source: Aging Cell. 2020 Sep 10;19(10):e13233. doi: 10.1111/acel.13233 (PMC7576303; doi:10.1111/acel.13233)
Supplement: Supplementary file 4 [file ACEL-19-e13233-s004.docx]

**SUPPLEMENTARY FIGURE LEGENDS**

**Figure S1. Brain slices from Tg-FDD mice shows normal excitatory LTP.** **(A)** Representative field excitatory postsynaptic potential (fEPSP) traces obtained from Hippocampal Schaffer collateral-CA1 from WT (circle) and Tg-FDD mice (square), showing the average baseline fEPSP (1-10 min.) and average fEPSP of the final ten min. of recording (40-50 min.) for each genotype. **(B)** Time course graph shows the presence of LTP on WT and Tg-FDD mice. **(C)** Data show average of normalized fEPSP slope for final ten min. of recording (40-50 min.) relative to ten min. baseline average. Data are mean ± SEM. P= 0.6037, t15=0.5302, Unpaired Student’s t test; n=6 slices from 2 WT mice, n=11 slices from 3 Tg-FDD mice.

**Figure S2. Synaptic localization of excitatory synaptic markers in Tg-FDD mice is normal**. **(A)** Double immunofluorescence using anti-Synapsin-1 (red) and anti-GluN1 (green) in brain sections of Tg-FDD and WT mice. **(B)** Quantification of the merge of both markers in cortex and hippocampus. P < 0.05 indicated on each graph, Mann-Whitney test, n = 9 photographs from 3 different animals per genotype, Data represented as mean ± SEM. Scale bar 10 μm.

**Figure S3. GABAergic synapse pathway in CAA RNA-Seq data set. (A)** Enriched pathway in upregulated genes in CAA vs normal brain temporal cortex region. Differential expression cutoff of fold change >1.2, FDR adjusted p value <0.05. **(B)** Genes upregulated in CAA in GABAergic synapse pathway.
